# The Preparation and Properties of Multilayer Cu-MTa_2_O_5_ Composite Coatings on Ti6Al4V for Biomedical Applications

**DOI:** 10.3390/nano9101498

**Published:** 2019-10-21

**Authors:** Zeliang Ding, Yi Wang, Quan Zhou, Ziyu Ding, Yiyong Wu, Yuefang Zhu, Wensong Shi, Quanguo He

**Affiliations:** 1 School of Mechanical Engineering, Hunan University of Technology, Zhuzhou 412007, China; wy15292222379@163.com (Y.W.); zhouquan321@163.com (Q.Z.); 2 School of Packaging and Materials Engineering, Hunan University of Technology, Zhuzhou 412007, China; dingziyu0320@163.com; 3 School of Life Sciences and Chemistry, Hunan University of Technology, Zhuzhou 412007, China; wyy5082010@163.com; 4 Zhuzhou Institute of Food and Drug Control, Zhuzhou 412008, China; hunandexiaozhu@163.com (Y.Z.); swshn@21cn.com (W.S.)

**Keywords:** copper, Ta_2_O_5_, ceramic coating, Ti6Al4V, magnetron sputtering

## Abstract

For the enhancement of the anticorrosion and antibacterial performance of the biomedical alloy Ti6Al4V, a novel Cu incorporated multilayer Ta_2_O_5_ceramic composite coating Cu-Ta_2_O_5_/Ta_2_O_5_/Ta_2_O_5_-TiO_2_/TiO_2_/Ti (coating codeCu-MTa_2_O_5_) was developed by radio frequency (RF) and direct current (DC) reactive magnetron sputtering. Meanwhile, to better display the multilayer Ta_2_O_5_ coating mentioned above, a monolayer Ta_2_O_5_ ceramic coating was deposited onto the surface of Ti6Al4V alloy as a reference. The surface morphology, microstructure, phase constituents, and elemental states of the coating were evaluated by atomic force microscopy, scanning electron microscopy, X-ray diffraction, and X-ray photoelectron spectroscopy, respectively. The adhesion strength, wettability, anticorrosion and antibacterial properties of the coating were examined by a scratch tester, contact angle measurement, electrochemical workstations, and plate counting method, respectively. The results showed that the deposited coatings were amorphous and hydrophobic. Cu doped into the Ta_2_O_5_ coating existed as CuO and Cu_2_O. A Ta_2_O_5_-TiO_2_/TiO_2_/Ti multi-interlayer massively enhanced the adhesion strength of the coating, which was 2.9 times stronger than that of the monolayer Ta_2_O_5_coating. The multilayer Cu-MTa_2_O_5_ coating revealed a higher corrosion potential and smaller corrosion current density as compared to the uncoated Ti6Al4V, indicating the better anticorrosion performance of Ti6Al4V. Moreover, a 99.8% antibacterial effect of Cu-MTa_2_O_5_ coated against *Staphylococcus aureuswas* obtained.

## 1. Introduction

Ti6Al4V titanium alloy, as a medical implant material, has been commonly applied in dentistry, orthopedics, and other fields due to its excellent corrosion resistance, mechanical properties, and biocompatibility [1,2,3,4]. However, clinical studies have indicated that Ti6Al4V titanium alloy-related implants would be corroded by body fluids, releasing toxic and side-effectmetal ionssuch as V^5+^ and Al^3+^ [5,6], and causing local immune dysfunction [7], inflammation, and cytotoxic reactions in the human body [8]. During operations, bacteria will adhere to the surface of the implant, then propagate and form biofilms, resulting in postoperative infection, and in severe cases resulting in amputation and even death [9,10,11]. Moreover, the Ti6Al4V alloy itself has no bactericidal activity, but the titanium alloy with a vanadium-rich phase can even attract the adhesion of bacteria [12]. Therefore, its application in clinical research on improving the anticorrosion and antibacterial performance of Ti6Al4V alloy has become a significant challenge.

Coatings, representing an effective method for the surface functionalization of materials, can endow the surface of materials with various properties as required, such as corrosion resistance [13], electroconductibility [14,15,16,17,18], selectivity [19], antibacterial properties [20], and biocompatibility [21]. In recent years, bioactive ceramics, such as TiO_2_ [22], ZrO_2_ [23], MgO [24], HA [25], Nb_2_O_5_ [26], and tantalum pentoxide (Ta_2_O_5_), have served as modified coatings on the Ti6Al4V surface, among which the Ta_2_O_5_ ceramic coating exhibits excellent comprehensive properties in terms of anticorrosion, antiwear and biocompatibility. Xu et al. [27,28] prepared a Ta_2_O_5_ coating on the Ti6Al4V surface with double-cathode plasma sputtering deposition technology. Results indicated that the coated Ti6Al4V alloy showed higher corrosion potential and lower corrosion current density, and remarkably enhanced the adhesion and proliferation activity of mouse embryonic fibroblasts compared to an uncoated Ti6Al4V alloy. Hu et al. [3] found that the hardness and elastic modulus of a Ta_2_O_5_-coated Ti6Al4V alloy was 4-fold and 2-fold higher than that of uncoated Ti6Al4V, respectively. Moreover, the wear rate of the Ta_2_O_5_ coating (10^−6^ mm^3^·N^−1^·m^−1^) was reduced by approximately two orders of magnitude compared to the uncoated Ti6Al4V under the dry sliding wear condition with 2.3–5.3N loads.However, owing to the mismatch in properties, such as the modulus of elasticity (MOE) and thermal expansion coefficient (CTE), between Ta_2_O_5_ ceramic coatings and Ti6Al4V alloy substrates, the bonding between them is usually poor. Rahmati et al. [1,29] found that the bonding force between a Ta_2_O_5_ coating prepared by reactive sputtering and Ti6Al4V was 713 mN, which rose to 1907 mN and 2500 mN, respectively, after heat treatment and preparation parameters optimization. The low adhesion of the coating to the substrate can cause the coating to fall off the surface of the substrate during surgery or service, which can lead to rejection of the implant and side effects on the surrounding tissue, and ultimately lead to the implant’s failure [30]. Generally, the bonding force of the coating needs to be more than 30N to meet the needs of engineering applications [31]. Therefore, this creates hurdles for the clinical applications of a Ta_2_O_5_ coating. 

Studies have verified that the introduction of an intermediate layer between coatings and substrates can reduce the mismatch degree of properties among different materials and improve the bonding properties of the coating/substrate system. In recent years, not only has a monolayer of Ti [32], TiO_2_ [33], ZrO_2_ [34], SiC [35], or TiN [36] been added as an intermediate layer between bioactive ceramic and Ti6Al4V alloy, but also bilayers such as TiN/TiO_2_ [37], ZrN/Zr [38], and TiN/Ti [39] have been used as the intermediate layer. These intermediate layers not only improve the bonding strength of the ceramic coating to the Ti6Al4V alloy substrate, but also enhance the mechanical properties, wear resistance, and corrosion resistance of the coated Ti6Al4V. However, limited literature exists about the addition of an intermediate layer between the Ti6Al4V alloy substrate and a Ta_2_O_5_ ceramic coating deposited by RF magnetron sputtering.

It is known that copper is one of the most effective antibacterial additives, with a broad antibacterial spectrum and low cytotoxicity [40,41]. Not only does it have a potent bactericidal effect on Gram-positive and Gram-negative bacteria, but it can also effectively treat postoperative bacterial infections [11]. At the same time, copper, as an indispensable trace element in the human body, plays a vital role in maintaining the normal physiological ability of the body [42], such as the formation of osteoblasts in bone metabolism [43], regulating microvascular development, and accelerating skin wound healing [44,45]. Missing copper ions in the body may result in impaired bone growth and bone strength in animals [46]. Copper, as a modifier, can be appropriately used for eliminating bacterial infection on medical implant materials as far as the above advantages are concerned. A study by Zhang et al. [47] reported that the number of *Staphylococcus aureus* on the surface of a TiO_2_ coating doped with Ca and P elements was 16 × 10^5^ cm^−2^, while doping with Cu elements led to a decrease of 81.9%. Chan et al. [48] reported a nearly 40% antibacterial rate of an a-C:H coating on *Escherichia coli*, while 99.9% of the antibacterial rate of Cu/a-C:H coating was also reported. Also, Rosenbaum et al. [49] demonstrated that titanium had no antibacterial activity, while the antibacterial rate of a Cu-TiO_2_coating on *E. coli* and *S. aureus* can reach 100%. Unfortunately, the doped copper may reduce the corrosion resistance of the copper-bearing coating in chloride media because of the release of the Cu ions, leading to the premature failure of the coating [50,51].

Based on the above situation, this paper aims at improving the anticorrosion and antibacterial properties of a Ti6Al4V titanium alloy medical implant, whereby a novel copper doped multilayer tantalum oxide composite coating Cu-Ta_2_O_5_/Ta_2_O_5_/Ta_2_O_5_-TiO_2_/TiO_2_/Ti (coating code Cu-MTa_2_O_5_) was deposited on the Ti6Al4V substrate by magnetron sputtering. The surface morphology, microstructure, and phase composition of the coating were analyzed by scanning electron microscopy (SEM), atomic force microscopy (AFM), X-ray diffraction (XRD), energy-dispersive spectroscopy (EDS), and X-ray photoelectron spectroscopy (XPS). The wettability, bonding strength, corrosion resistance, and antibacterial property of the coating were determined by a contact angle measuring instrument, scratch tester, electrochemical workstation, and plate counting method, respectively. For comparison, these investigations were also performed on monolayer Ta_2_O_5_ coating samples. The findings from this study are expected to provide a valuable reference for the surface modification of titanium alloy implant materials.

## 2. Material and Experimental Methods

### 2.1. Coatings Deposition

**Figure 1:**
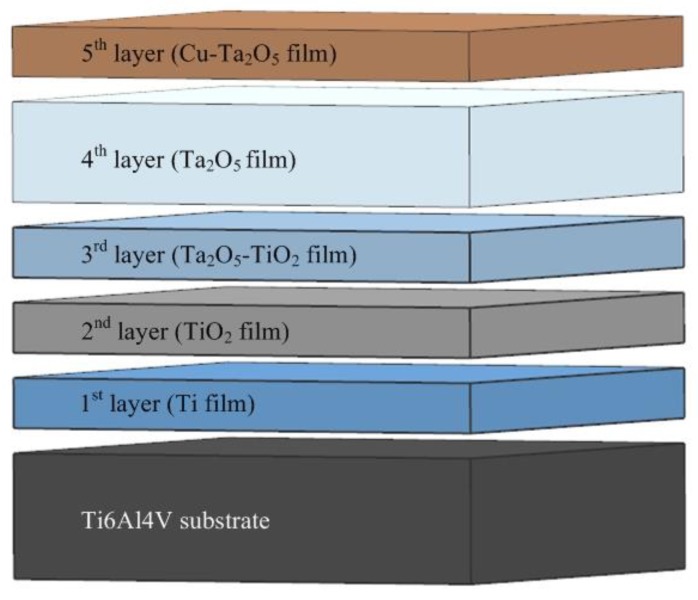
Schematic representation of Cu-MTa_2_O_5_ multilayer composite coating structure.

Figure 1 depicts the structures of the Cu-MTa_2_O_5_ coating. As shown in Figure 1, the Cu-MTa_2_O_5_coating contains five layers of film, wherein the first to third layers are a Ti film, TiO_2_ film, and TiO_2_-Ta_2_O_5_ composite film, respectively. These three films serve as the interlayer, in which elements of adjacent layers penetrate each other in reducing the interface stress and enhance the bonding property of the coating. The fourth layer of the Ta_2_O_5_ ceramic film is an anticorrosion layer, and the fifth layer (outer layer) is Ta_2_O_5_ doped with Cu, which has an antibacterial effect. 

A Ti6Al4V alloy (BAOTI Group Co., Ltd., Baoji, Shanxi, China) with a size of 10 mm × 10 mm × 0.6 mm and silicon wafers (10 mm × 10 mm × 2 mm) were used as substrates. The chemical compositions of the Ti6Al4V alloy are shown in Table 1. The Ti6Al4V substrates were mechanically ground using SiC sandpaper (240–2000 grit) and polished with aqueous alumina solution to gain a surface roughness of 5 nm. The silicon wafer and polished Ti6Al4V were ultrasonically cleaned in acetone and ethanol for 15 min, respectively, and vacuum-dried.

A JCP-450 high-vacuum magnetron sputtering coating system (BJTN, Beijing, China) was employed for plasma cleaning and coating deposition. Before deposition, the substrates and targets were separately cleaned by plasma cleaning for 20 min to remove the surface contaminants and improve the surface activity. Afterward, Cu-MTa_2_O_5_coatings were prepared on the substrates by successively depositing a Ti metal film, TiO_2_ ceramic film, Ta_2_O_5_-TiO_2_ ceramic composite film, Ta_2_O_5_ ceramic, and Cu-Ta_2_O_5_ composite film. Cu-MTa_2_O_5_ coatings deposited on Si substrates were employed to characterize the microstructure of the cross-section of the coating, while those on Ti6Al4V substrates were used to evaluate properties such as adhesion strength, wettability, corrosion resistance, and antibacterial behavior. The purity of the Ta_2_O_5_, Ti, and Cu targets (ZNNM, Beijing, China) with a size of φ75 mm × 5 mm was 99.99%. Argon and oxygen with 99.99% purity were used as the sputtering gas and reaction gas, respectively. A monolayer Ta_2_O_5_ coating was prepared on Ti6Al4V substrate as a control. Table 2 lists the details of the coating preparation parameters. 

### 2.2. Coatings Characterization 

The crystal structure of the coatings was investigated by XRD (Ultima IV, Rigaku Corporation, Tokyo, Japan) analysis. The surface and fractured cross-sectional morphology, elemental composition, and map of the coatings were characterized by SEM (SU8000, Hitachi Group, Tokyo, Japan) equipped with EDS. The roughness of the coated surface was detected with AFM (EasyScan2, Nanosurf, Basel, Switzerland). The elemental chemical states in Cu-MTa_2_O_5_ coatings were determined by XPS (EscaLab 250Xi, Thermo Fisher Scientific Inc., Waltham, MA, US).

### 2.3. Scratch Test

The scratch test is the most common method to quantitatively evaluate the adhesion strength of a coating [36]. In this work, MFT-4000 multifunctional surface test equipment (LICP, Lanzhou, China) was employed to investigate the bonding strength of a coating deposited on the surface of a Ti6Al4V alloy. During the measurement, a diamond indenter with a radius of 200 μm was applied to the coating surface with a normal load ranging from 0 to 20 N and moved with a loading rate of 10 N/min and a scratch length of 6.0 mm. Meanwhile, the changes of the acoustic emission, friction force, friction coefficient, and normal force with the scratch distance were continuously recorded. The critical load (L_c_) was determined by comprehensively analyzing the changes in friction force and microscopic observation. Two critical loads of L_c1_ and L_c2_ were used to characterize the failure mode of the coating. L_c1_ denotes the load required for initial chipping in the scratch track or next to the scratch track, while L_c2_ denotes the load required for complete failure and represents the coating adhesion strength [52]. To determine the failure position and failure mode of the coating, an ultra-depth of field optical microscope (KH-7700, Seika Corporation, Tokyo, Japan) was used to observe the morphology of the scratch. 

### 2.4. Contact Angle Measurements

The water contact angle is usually used to evaluate the wettability of the material [53]. The contact angles of uncoated and coated Ti6Al4V alloy were detected by a JC20001 contact angle goniometer (POWEREACH, Shanghai, China) with the sessile drop method (using a 2-μL distilled water droplet). The droplet images were obtained by the optical system of the goniometer and the contact angle was measured from the images.

### 2.5. Electrochemical Experiments

Electrochemical experiments were performed using an SP-15/20A electrochemical workstation (Bio-logic Scientific Instruments, Seyssinet-Pariset, France) with a typical three-electrode configuration. A saturated Ag/AgCl electrode was used as the reference electrode (RE), and a platinum sheet was used as the counterelectrode (CE). The uncoated and coated Ti6Al4V were employed as the working electrodes (WE), which were fully coated with epoxy resin, except for a 1-cm^2^ area for exposure to SBF solution. Simulated body fluid (SBF) electrolyte with a 7.4 pH was used [54]. Potentiodynamic polarization curves were recorded using a scanning rate of 1 mV/s from −0.3 V to +2.0 V. The corrosion potential (E_corr)_ and corrosion current density (I_corr_) were obtained from the polarization curves using the Tafel extrapolation method. 

### 2.6. Antibacterial Tests 

The plate counting technique is usually employed for investigating the antibacterial activity of modified coatings [55]. Gram-positive *S. aureus* is one of the most common pathogens causing biomaterial-centered infection (BCI) and peri-implant inflammation [56]. In this study, *S. aureus* strain ATCC 6538 obtained from Guangzhou Industrial Microorganism Testing Center (Guangzhou, China) was used to evaluate the antibacterial activity of the coatings with the plate counting method. Before the tests, all the specimens were sterilized at 121 °C, 0.1 MPa for 30 min. A sterilized 0.9% NaCl solution was used to modulate the concentration of the bacterial suspension. 

The sterilized sample was placed in a centrifuge tube containing 10^7^ CFU/mL bacterial suspension of 4 mL and incubated at 37 °C for 24 h. After incubation, the specimen was taken out and the bacterial suspension was fully mixed by a mixer. Then 100 μL of the mixed bacterial suspension were inoculated in Luria‒Bertani (LB) agar plates and cultivated at 37 °C for 24 h. Finally, the bacterial colonies grown were counted by an automatic colony detection instrument (Sphere Flash, Barcelona, Spain). The antimicrobial rate (*P*) was obtained by Equation (1) [40]:

(1) $$P=\frac{X-Y}{X}\times 100\%$$

where *X* and *Y* are the number of living bacteria observed on the uncoated and coated Ti6Al4V, respectively. 

### 2.7. Statistical Analysis

Each experiment was carried out in triplicate and the result is expressed as mean ± standard deviation (SD). To investigate the statistical significance between various sample groups, one-way analysis of variance was applied and *p* < 0.05 was regarded as statistically significant. 

## 3. Results and Discussion

### 3.1. Microstructural Characterization

**Figure 2:**
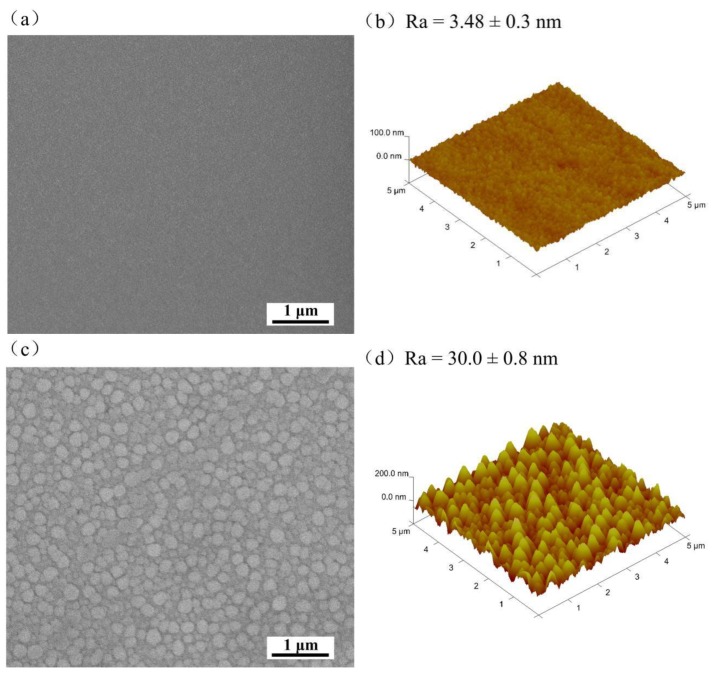
SEM micrographs of (**a**) Ta_2_O_5_ coating and (**b**) Cu-MTa_2_O_5_coating. AFM images of (**c**) Ta_2_O_5_ coating and (**d**) Cu-Ta_2_O_5_ coating.

The surface topographies of the coated Ti6Al4V samples are displayed in Figure 2. The surface of the Ta_2_O_5_ coating was flat, smooth, and the grain size was uniform (Figure 2a). In contrast, the grain of the Cu-Ta_2_O_5_ coating surface (Figure 2c) is larger (10–100 nm), with clear grain boundariesand larger grain gaps, indicating the lower compactness of the outer layer. The increase in the grain size of the Cu-MTa_2_O_5_ coating relates to the doping amount of Cu [57]. Three-dimensional AFM topographies with an area of 5 μm × 5 μm showed that the main features of both coating surfaces were characterized by peak-type particles (Figure 2b,d). The particles size of Ta_2_O_5_ coating was small, with an average roughness Ra value of 3.48 ± 0.3 nm, while the roughness of the Cu-MTa_2_O_5_ coating was 30 ± 0.8 nm. A low element doping amount in the coating can increase the compactness of the coating and reduce the surface roughness, while a high doping amount increases the surface roughness [58].

**Figure 3:**
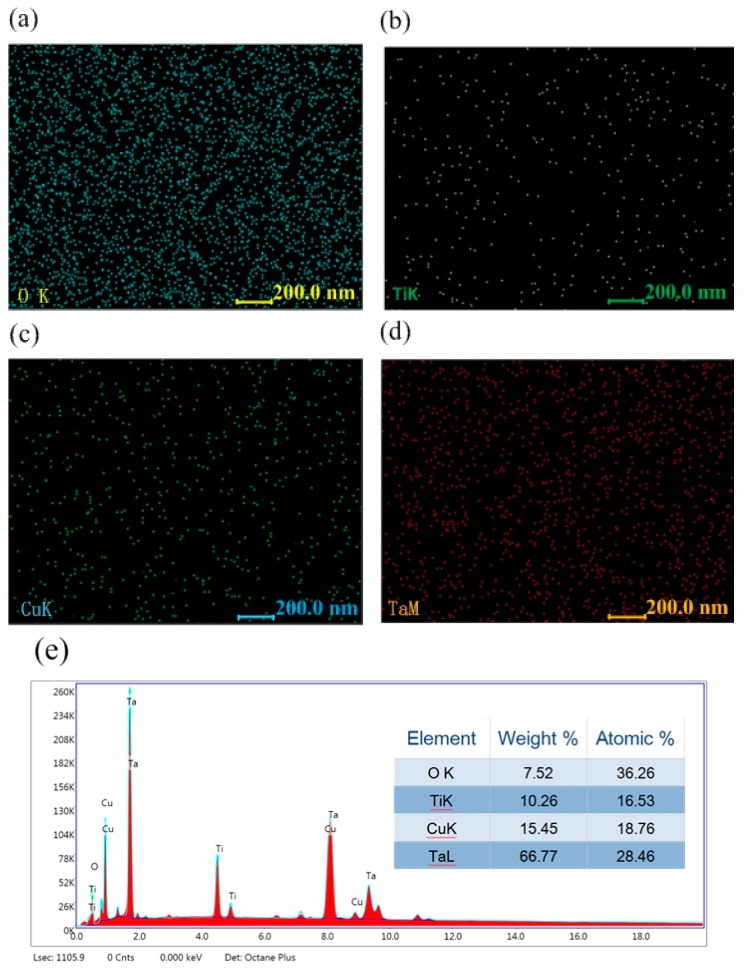
EDS elemental image of the Cu-MTa_2_O_5_ coating surface: element mapping (**a–d**); element content (**e**).

Figure 3 shows the EDS analysis results of the surface Cu-MTa_2_O_5_ coating. As shown in Figure 3a–d, O, Ti, Ta, and Cu were uniformly distributed on the coating surface, indicating that the copper element was incorporated into the Ta_2_O_5_ film and the Ti element diffused to the outer layer of the coating. It can be seen from Figure 3d that the atomic percentages of O, Ti, Ta, and Cu in the Cu-MTa_2_O_5_ coatings were about 36.26%, 16.53%, 28.46%, and 18.764%, respectively. The element content in the coating mainly connects with the preparation parameters such as sputtering power, oxygen flow rate, and ratio of oxygen to argon.

**Figure 4:**
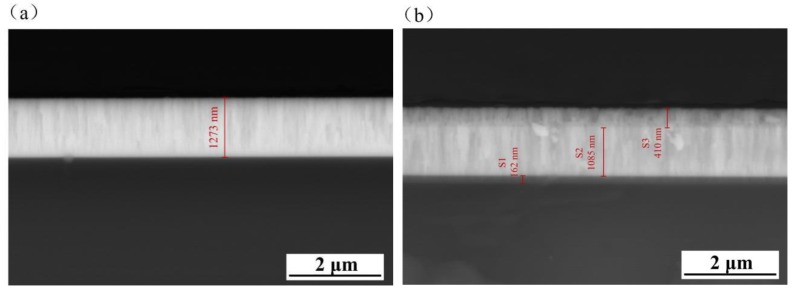
The cross-sectional SEM of the coated Ti6Al4V samples of (**a**) Ta_2_O_5_ and (**b**) Cu-MTa_2_O_5_.

Figure 4 displays the cross-sectional characteristics of the coating samples. The bonding interface between the Ta_2_O_5_ monolayer coating and the Ti6Al4V substrate was clearly observed, and the coating thickness was about 1273 nm (Figure 4a), while the cross section of the Cu-MTa_2_O_5_ coating shows three distinct regions (Figure 4b), namely S1, S2, and S3, with corresponding thicknesses of 162 nm, 1085 nm, and 410 nm, respectively. Depending on the structure (Figure 1) and preparation parameters (Table 2) of the Cu-MTa_2_O_5_ coating, it was deduced that the S1, S2, and S3 regions corresponded to the Ti film, TiO_2_-Ta_2_O_5_/Ta_2_O_5_ film, and Cu-Ta_2_O_5_ film, respectively. In the S2 region, there was no observable interface between the TiO_2_-Ta_2_O_5_ film and Ta_2_O_5_ film, and there were no microspores and cracks observed. This contributes to the lowering of the interfacial stress and improves the adhesion of the coating. In addition, the density of the S3 region was significantly lower than that of the S2 region, which was related to a large amount of Cu incorporation. 

**Figure 5:**
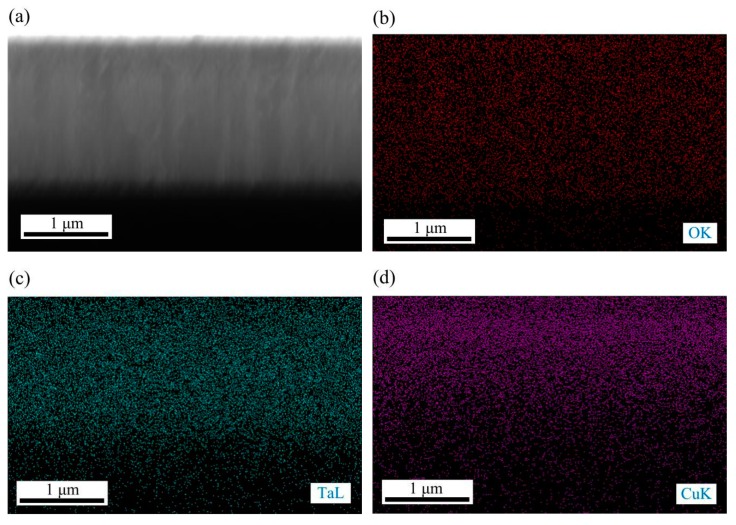

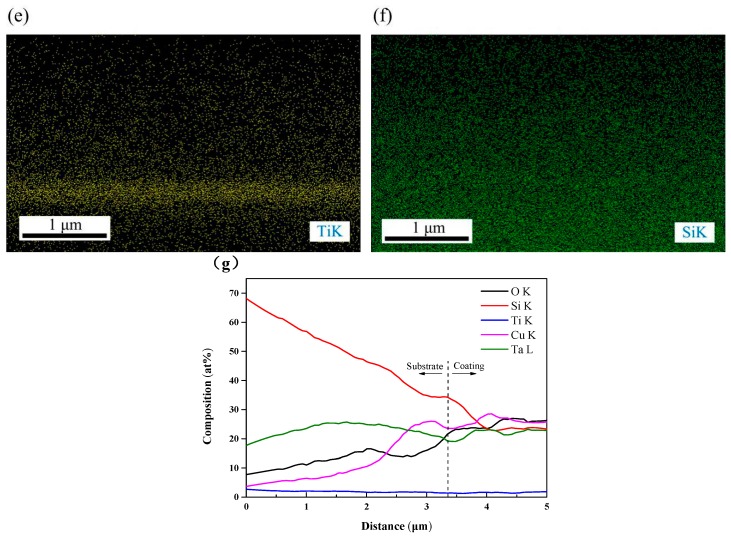
Cross-sectional SEM and EDS measurement of the Cu-MTa_2_O_5_ coating on the Si substrate: (**a**) SEM, (**b**–**f**) EDS mapping and (**g**) EDS line scan profile along with the thickness of the coated sample.

Figure 5 presents the EDS detection results of the cross section of the Cu-MTa_2_O_5_ coating sample. As shown in the figure, the Ta, Cu, and O elements showed a uniform distribution in the coated sample, while their concentration gradually increased far from the coating‒substrate interface. All elements (Ta, Cu, Ti, and O) were not only distributed throughout the coating, but also diffused into the substrate. Elemental diffusion between adjacent layers helps form a metallurgical bonding interface, reducing interfacial stress and thereby increasing the coating bond strength.

**Figure 6:**
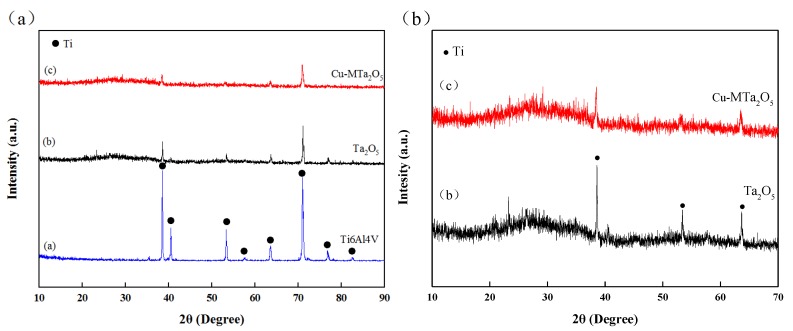
XRD patterns of all samples (**a**) and coating samples (**b**).

Figure 6 shows the XRD spectrum of the uncoated and coated Ti6Al4V specimens. There were no characteristic peaks of the Cu and Ta_2_O_5_ in the XRD spectrum of the coating (Figure 6a), indicating that the Cu and Ta_2_O_5_ in the coating are amorphous structures [59]. The appearance of an amorphous structure relates to factors such as low deposition temperature and small sputtering power [60,61]. The previous study found that a Ta_2_O_5_ film deposited at room temperature begins to crystallize above an annealing temperature of about 800 °C [62], while the crystallization temperature of the Cu film was above 300 °C [20].

Additionally, the diffraction peaks of Ti appeared in the XRD pattern of the coatings, which may be the diffusion of the Ti from the intermediate layer or the substrate due to the porous structure and small thickness of the coating. Compared with the Ta_2_O_5_ coating, the Cu-MTa_2_O_5_ coating had a bigger thickness and weaker Ti peak strength. Analyzing the XRD high-resolution diagram (Figure 6b), a hump in the range of 20–40°of 2θ was observed, and its position was consistent with the interval diffraction peak of the Ta_2_O_5_ (JCPDS card No. 25-0922). The chemical valence of the elements in the coating should be further determined using XPS.

**Figure 7:**
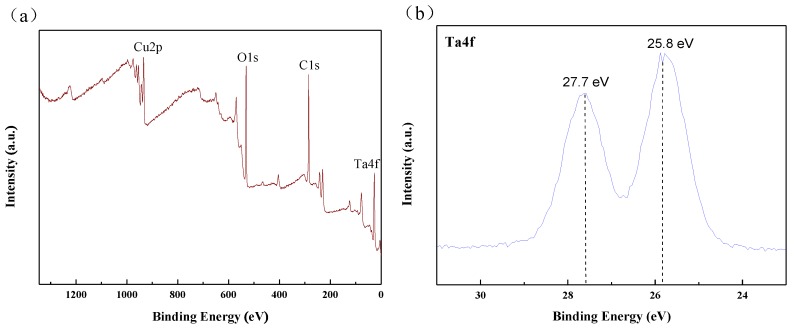

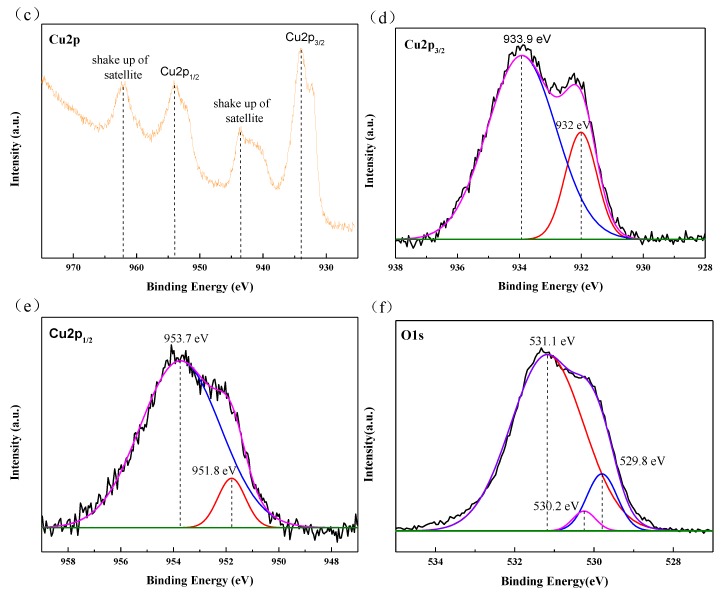
(**a**) XPS survey spectrum and (**b**) Ta4f, (**c**) O1s, (**d**–**f**) Cu2p high-resolution spectra of the Cu-MTa_2_O_5_ coating.

Figure 7 shows the XPS spectrum of the Cu-MTa_2_O_5_ coating. As shown in Figure 7a, the Ta 4f, O 1s, and Cu 2p spectra appear in the XPS survey spectrum of the coating, revealing the existence of O, Cu, and Ta in the Cu-MTa_2_O_5_ coatings.The Ta4f spectrum displayed in Figure 7b contains two peaks at the binding energies of 25.8 eV and 27.7 eV, corresponding to Ta 4f_7/2_ and Ta 4f_5/2_, respectively, indicating that Ta exists in the Cu-MTa_2_O_5_ coating in the form of Ta_2_O_5_, which is consistent with a previous report [63]. 

Figure 7c shows the XPS spectrum of the Cu 2p that was used to analyze the chemical bonds of Cu species. It was observed that the broad Cu 2p_3/2_ and Cu2p_1/2_ peaks can both be deconvoluted into two peaks, as shown in Figure 7d,e, respectively. In the Cu2p_3/2_ spectrum, the peak at 933.9 eV relates to CuO, while the peak at 932 eV relates to Cu or Cu_2_O [49]. In the deconvolution diagram of Cu 2p_1/2_ (Figure 7e), the 951.8 eV peak comes from copper or Cu_2_O, while the 953.7eV peak comes from CuO. Furthermore, two other shake-up peaks belong to CuO [46], which further confirms the presence of CuO in Ta_2_O_5_ [64]. The doped Cu exists in the Cu-MTa_2_O_5_ coating in the oxidation state of copper because copper can easily be oxidized [65].

The high-resolution XPS spectrum of O1s, shown in Figure 7c, contains three deconvoluted peaks. The large peak with the binding energy of 531.3 eV was assigned to Ta_2_O_5_ [66], while the peaks with a binding energy of 530.2 eV and 529.8 eV correspond to Cu_2_O and CuO, respectively [67,68]. The above results indicate that Ta exists in the Cu-MTa_2_O_5_ coatings in the form of Ta_2_O_5_, while Cu exists in CuO and Cu_2_O. Ta_2_O_5_ affects the enhancement of the anticorrosion and biocompatibility of the metal implant materials [3,28], while Cu plays an important role in enhancing the antibacterial activity of the Cu-MTa_2_O_5_ coating [69].

### 3.2. Adhesion Strength

**Figure 8:**
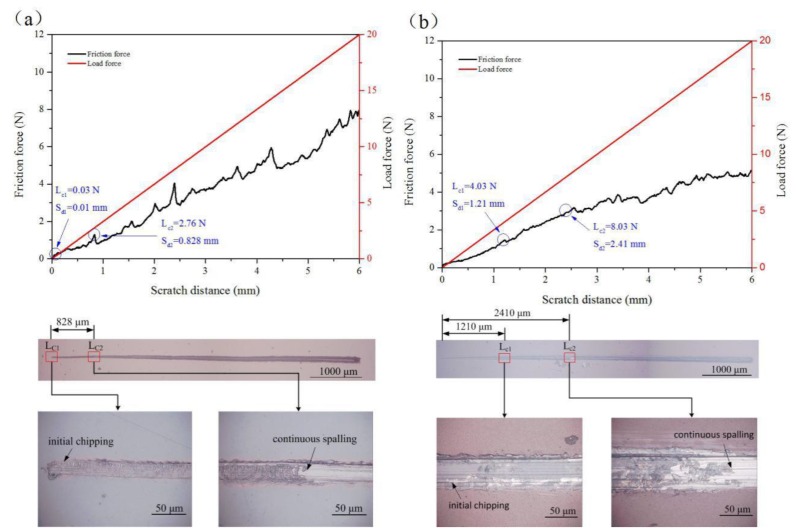
Scratch curves and scratch track images of (**a**) Ta_2_O_5_ coating and (**b**) Cu-MTa_2_O_5_ coating.

Figure 8 displays the scratch curves and scratch track images of the scratch test. As shown in Figure 8a, the initial chipping appeared on the Ta_2_O_5_ coatings at a distance of 0.01 mm with a corresponding load (L_c1_) of 0.03 N. When the diamond indenter moved 0.828 mm, the Ta_2_O_5_ coating completely fell off with an applied load of 2.76 N, indicating that the critical load L_c__2_ of Ta_2_O_5_ was 2.76 N. Figure 8b shows that the critical loads L_c1_ and L_c2_ for initial fragmentation and continuous spalling were 4.03 N and 8.03 N, respectively.Repeated measurements were taken at three different locations of each sample, and finally the adhesion strength of Ta_2_O_5_ and Cu-MTa_2_O_5_ was obtained: 1.0–3.5 mN and 7.0–9.0 mN, respectively.

The bonding mechanism between the coating and the substrate is complicated, and the bonding strength can be affected by many factors, such as the surface state of the substrate, preparation parameters, substrate temperature, intermediate layer, coating thickness, post-heat treatment [1,4,29,70], etc. For a multilayer Cu-MTa_2_O_5_ coating, its adhesion strength is higher than that of the single layer Ta_2_O_5_ coating due to the introduction of the multiple intermediate layers of Ta_2_O_5_-TiO_2_/TiO_2_/Ti. This is mainly because the element content in the Cu-MTa_2_O_5_ coating sample gradually changed from the Ti6Al4V substrate to the outer layer of the coating after the addition of the intermediate multilayer, which reduces the performance mismatch and interfacial stress between the Ta_2_O_5_ coating and the Ti6Al4V substrate, and enhances the bonding strength of the coating. Of course, the bonding strength of the Cu-MTa_2_O_5_ coating is not sufficient for clinical applications. In the future, it will be further improved by optimizing the preparation parameters, increasing the coating thickness, and performing post-heat treatment.

### 3.3. Wettability

**Figure 9:**
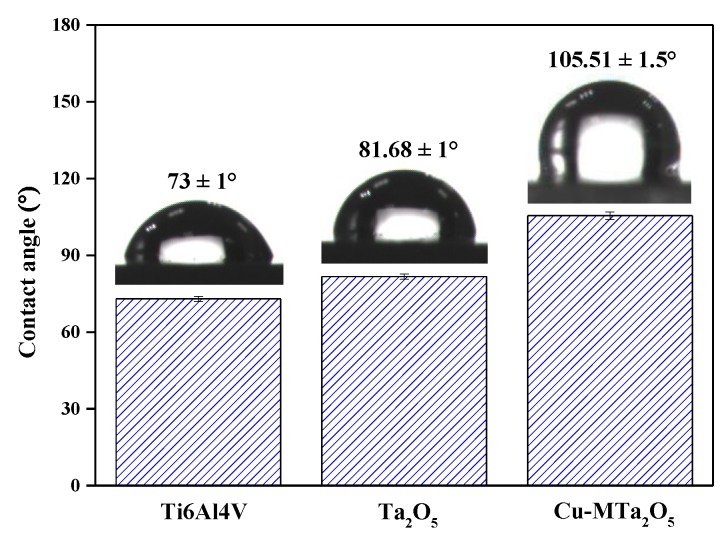
Contact angle measured for the uncoated and coated Ti6Al4V samples.

Wettability is one of the important surface properties that affect the response of cells/bacteria to implant materials [71]. The wettability of the surface of the material will be better and the biological activity will be higher due to the smaller contact angle [72]. Figure 9 shows the water contact angle data and water droplet images on uncoated Ti6Al4V, Ta_2_O_5_, and Cu-MTa_2_O_5_ coated Ti6Al4V. The uncoated Ti6Al4V showed a contact angle of 73 ± 1°, indicating a hydrophilic surface. The coated sample had a larger contact angle than the uncoated samples, wherein the Cu-MTa_2_O_5_ coating sample exhibited the largest contact angle (105.5 ± 1.5°), showing hydrophobicity. It is well known that the greater the roughness of the material surfaces, the greater the water contact angle [73]. The surface roughness value of the Cu-MTa_2_O_5_coating was about 8 times larger than that of the Ta_2_O_5_ coating (Figure 2), and thus exhibits a larger contact angle. For medical implant materials, a hydrophobic surface is considered helpful in stopping early bacterial adherence and forming microbial membranes, thus improving the anticorrosion properties of the materials [74].

### 3.4. Corrosion Behavior

**Figure 10:**
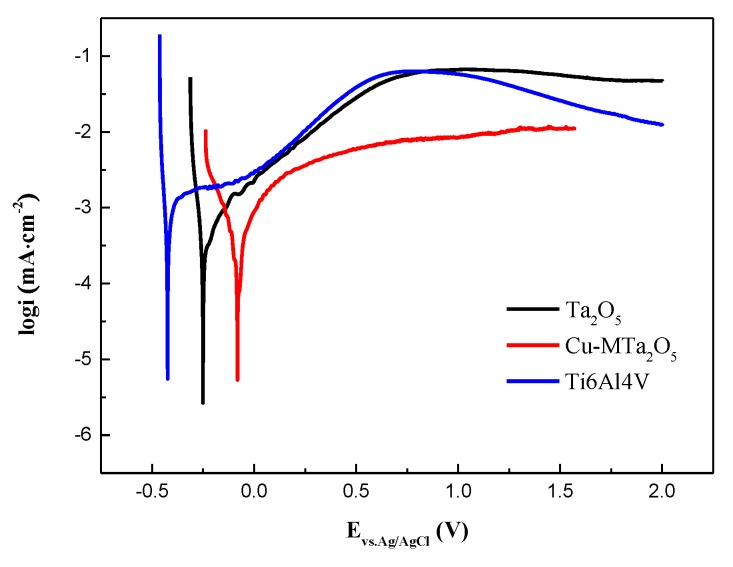
Potentiodynamic polarization curves of the coated specimens and Ti6Al4V.

Anticorrosion property is one of the critical indicators to evaluate the safety of metal implant materials. Figure 10 displays the potentiodynamic polarization curves acquired from uncoated Ti6Al4V and the coating specimens in SBF. The corrosion parameters such as corrosion potential (E_corr_) and corrosion current density (I_corr_) were derived by Tafel extrapolation of the polarization curves and listed in Table 2. The E_corr_ expresses the tendency to corrode and the I_corr_ shows the corrosion rate [75]. The E_corr_ of the Ti6Al4V was about −0.42 V vs. Ag/AgCl in SBF, while the values of Ta_2_O_5_ and Cu-MTa_2_O_5_ coating specimens moved toward the positive potential, which was −0.25 V and −0.08V, respectively, suggesting that corrosion of Ti6Al4V in SBF is retarded by the Ta_2_O_5_ coating and the Cu-MTa_2_O_5_ coating. The I_corr_ values of the Cu-MTa_2_O_5_ and Ta_2_O_5_ coating samples were 0.74 μA/cm^2^ and 0.48 μA/cm^2^, respectively, which are lower than those of Ti6Al4V (1.07 μA/cm^2^). These results signify that the I_corr_ of Cu-MTa_2_O_5_ and Ta_2_O_5_ coating samples is reduced about 1.5-fold and 2-fold, respectively, compared with the Ti6Al4V substrate in SBF. The polarization resistance R_p_ is usually used to evaluate the corrosion difficulty of materials [76]. As shown in Table 3, the R_p_ value of the coated sample was approximately 222% to 362% larger than that of Ti6Al4V. It is well accepted that high E_corr_, low I_corr_, and high R_p_ indicate the good anticorrosion property of the material [3]. Therefore, among the three samples, Ta_2_O_5_ coating samples show the best corrosion resistance, followed by Cu-MTa_2_O_5_ coating samples and uncoated Ti6Al4V titanium alloy. 

There are many factors that influence the corrosion behavior of materials, such as the composition and structure of materials, surface state, environmental conditions [77,78], etc. Compared with the bare Ti6Al4V samples, the improvement in corrosion resistance of the coated Ti6Al4V samples may be mainly attributed to the high stability of the Ta_2_O_5_ ceramic coating. Previous studies have shown that Ta_2_O_5_ films exhibit extreme resistance to dissolution over all combinations of pH and potential encountered in biomedical applications [79], effectively preventing corrosive ions from eroding the substrate. The reduction in corrosion resistance of Cu-MTa_2_O_5_ may be due in part to the low density of the outer Cu-Ta_2_O_5_ film (Figure 2b), which makes it easy for the corrosive medium to reach the surface of the Ti6Al4V substrate through the coating defects and corrode the substrate. On the other hand, Cu_2_O in the coating easily reacts with the chloride ions in the solution as follows [80]:

(2) $$Cu_{2}O+H_{2}O+4Cl^{-1}↔2CuCl_{2}+2OH^{-1}$$

The product CuCl_2_ of Equation (2) readily dissolves and releases Cu^2+^ ions, leading to the destruction of surface integrity and the reduction of the compactness of the coating, thus aggravating the corrosion of the substrate.

### 3.5. Antibacterial Property

**Figure 11:**
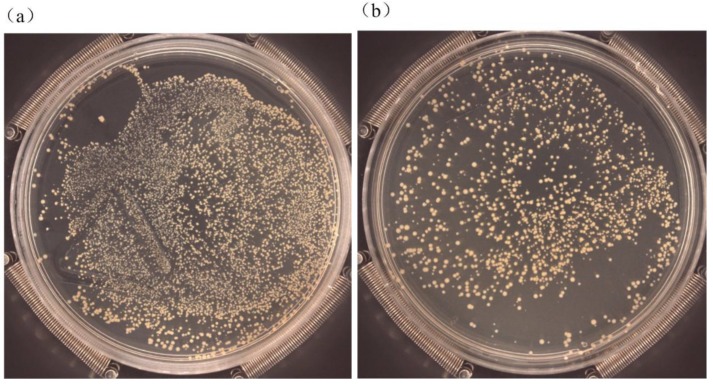

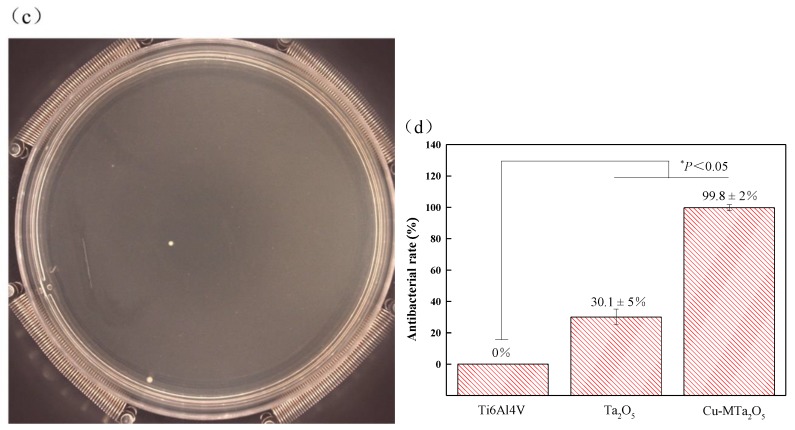
Petri dishes showing live *S. aureus* after being cultured at 37 °C for 24 h in LB agar containing: (**a**) Ti6Al4V, (**b**) Ta_2_O_5_ coating, and (**c**) Cu-MTa_2_O_5_ coating. (**d**) Antibacterial rates of Ti6Al4V, Ta_2_O_5_, and Cu-MTa_2_O_5_ coating against *S. aureus*.

The antibacterial properties of the Ti6Al4V alloy, Ta_2_O_5_, and Cu-MTa_2_O_5_ coatings against *S. aureus* colonies were investigated. Figure 11 showed the quantitative results of the live bacteria after 24 hours of culture at 37°C on LB agar plates. A large number of bacterial colonies were observed on the surface of Ti6Al4V- and Ta_2_O_5_-coated Ti6Al4V, while there were only two bacterial colonies on the Cu-MTa_2_O_5_ coating surface. The results showed that the Cu-MTa_2_O_5_ coating had strong antibacterial activity against *S. aureus* with an antibacterial rate of 99.8± 2%, while polished Ti6Al4V alloy did not possess antimicrobial activity.

The antibacterial activity of the Cu-MTa_2_O_5_ coating closely relates to the copper ions released from the coating surface [49]. When the coating was exposed to the bacterial solution, copper ions dissolved from the coating surface and diffused into the bacterial solution. Then, the copper ions were adsorbed on the bacterial cell wall by electrostatic action, limiting the bacterial activity and resulting in its metabolic disorder and death [81]. Moreover, after bacterial contact, copper ions pierced the cell membrane and entered the cell’s interior, destroying the integrity of the cell wall and causing bacterial death due to the loss of substances such as protein and reducing sugar [82]. In addition, copper ions entering the cells destroyed the respiratory chain of the bacteria, produced high levels of reactive oxygen species (ROS), degraded DNA, proteins and lipids, and eventually led to bacterial death [11,83]. Due to the complex mechanism of the bactericidal activity conferred by copper, the antibacterial activity of Cu-MTa_2_O_5_ coating may also come from the nanostructure of the coating surface, and its antibacterial mechanism may be a combination of various mechanisms, which need to be studied further.

The experimental results also showed that the Ta_2_O_5_ coating had a certain bactericidal ability, with an antibacterial rate of 30.1± 5%. The antibacterial activity of Ta_2_O_5_ coating was related to its amorphous structure [84] and the release of Ta^5+^ ions [85]. At present, there are very few studies on the antibacterial mechanism of Ta_2_O_5_, and detailed work needs to be carried out.

## 4. Conclusions

In summary, a multilayer composite coating with excellent antibacterial and anticorrosion properties was successfully deposited on a biomedical alloy, Ti6Al4V, using magnetron sputtering. The coatings, with a total thickness of about 1657 nm, were uniform, amorphous, and hydrophobic. The bond strength of the multilayer coating increased by 198% compared to the single-layer Ta_2_O_5_ coating. Moreover, the corrosion resistance and antibacterial properties of the Cu-MTa_2_O_5_ coating samples were significantly improved compared to the uncoated Ti6Al4V. Therefore, this study provides an alternative medical function coating for a titanium alloy surface. However, further work such as optimization of the preparation parameters, improvement of the bond strength, evaluation of the biocompatibility, in vivo experiments, etc., needs to be carried out.

## Figures and Tables

**Table 1:** Chemical composition of Ti6Al4V alloy (wt %).

Composition	Al	V	Fe	O	C	N	H	Ti
wt %	6.8	4.5	0.3	0.2	0.1	0.05	0.015	Balance

**Table 2:** Coating preparation parameters.

Coating Code	Layer Number	Coating Material		Sputtering Mode	Sputtering Power (W)	Deposition Time (min)	Gas Flow (sccm)	
							Ar	O_2_
Cu-MTa_2_O_5_	1st layer	Ti		DC sputtering	200	8	20	/
	2nd layer	TiO_2_		DC reactive sputtering	200	8	16	4
	3rd layer	Ta_2_O_5_-TiO_2_	Ta_2_O_5_	RF sputtering	200	8	20	5
			TiO_2_	DC reactive sputtering	200			
	4th layer	Ta_2_O_5_		RF sputtering	200	105	20	/
	5th layer	Cu-Ta_2_O_5_	Cu	DC sputtering	80	15	20	/
			Ta_2_O_5_	RF sputtering	200			
Ta_2_O_5_	/	Ta_2_O_5_		RF sputtering	200	120	20	/

**Table 3:** Corrosion parameters obtained from the polarization curves shown in Figure 10.

Specimen	Ti6Al4V	Ta_2_O_5_	Cu-Ta_2_O_5_
E_corr_ (V vs. Ag/AgCl)	−0.42 ± 0.02	−0.25 ± 0.01	−0.08 ± 0.01
I_corr_ (μA/cm^2^)	1.07 ± 0.021	0.48 ± 0.004	0.74 ± 0.005
β_a_ (mV/decade)	835.8 ± 15	373.9 ± 8	398.0 ± 4
β_c_ (mV/decade)	45.1 ± 4	51.1 ± 2	205.5 ± 4
R_p_ (Ω·cm^−2^)	17.3 ± 0.9	55.68 ± 1	80 ± 0.7
